# Towards Explainable Multimodal Sensing for Swimming Analysis: Early Findings from the SWIM-360 Project

**DOI:** 10.3390/s25227047

**Published:** 2025-11-18

**Authors:** Vanessa Camilleri, Reno Yuri Camilleri, Mark Fialovszky, Daniel Pace, Dylan Seychell, Matthew Montebello

**Affiliations:** Department of AI, University of Malta, MSD 2080 Msida, Maltamatthew.montebello@um.edu.mt (M.M.)

**Keywords:** artificial intelligence (AI), explainable artificial intelligence (XAI), multimodal sensing, swimming analysis, wearable sensors, sensor technology, video-based pose estimation, human–computer interaction

## Abstract

Swimming performance analysis increasingly depends on multimodal sensing systems that capture physiological and biomechanical signals in real-world aquatic environments. While progress has been made in sensor fidelity and automated analysis, the interpretability of these systems remains limited, constraining their uptake in coaching practice. This paper presents early findings from the SWIM-360 project, which investigates how explainable artificial intelligence (XAI) can support transparent and actionable insights for swimming performance. We report preliminary results from EO SwimBETTER and TrainRed sensors, together with proof-of-concept outputs from video-based pose estimation. In parallel, we introduce mock-up visualisations and interaction concepts designed to elicit coach feedback on requirements for explainability. A qualitative questionnaire with eight professional swimming coaches was conducted to elicit requirements for explainable feedback. Their responses informed the design of a multimodal, coach-centred explainability framework. Rather than providing a fully integrated model, the paper proposes a methodological framework that combines multimodal sensing with explainability-driven design principles. Our findings highlight both the feasibility and the challenges of translating sensor data into interpretable knowledge for athletes and coaches. By embedding explainability at the earliest design stage, this study proposes an explainability-driven design framework linking multimodal sensing and user requirements. These early findings highlight how XAI principles can guide the creation of trustworthy, coach-centred decision-support tools in aquatic sports.

## 1. Introduction

In recent years, sports science has made big steps forward through the use of wearable sensors, computer vision, and artificial intelligence (AI). Swimming is a good example, since performance depends on a mix of biomechanics, physiology, and technique. Tools such as inertial measurement units (IMUs) can measure stroke timing and turns, near-infrared spectroscopy (NIRS) can track muscle oxygen levels linked to fatigue, and video-based pose estimation can capture joint movement without markers. Together, these technologies provide much richer information than traditional stopwatches or coach observations.

The challenge is that the results from these systems are often difficult to understand. Many AI models act as “black boxes,” producing outputs that are not easy for coaches or athletes to interpret. Some research has focused mainly on improving accuracy [1,2], while others argue that clear explanations are essential for trust and real use in training [3,4]. Explainable AI (XAI) methods are beginning to address this gap. For example, SHAP (SHapley Additive Explanations) and LIME (Local Interpretable Model-agnostic Explanations) can show which features of sensor data influenced a prediction, while visual overlays and attention maps can highlight which body parts or motion phases the model used to classify a stroke. Still, in swimming, these approaches remain relatively underexplored.

This paper presents early results from the SWIM-360 project, which explores how multimodal sensing and explainability can work together in swimming. Given the small participant pool, this study adopts an exploratory scope and is designed to collect qualitative insights rather than establish generalisable findings. We report findings from IMU and NIRS sensors, including data collected using TrainRed near-infrared spectroscopy sensors (Train. Red B.V., Gelderland, The Netherlands) to monitor muscle oxygen saturation (SmO2) and EO-SwimBETTER IMU-based sensors (EO, Sydney, Australia) for biomechanical motion capture, alongside proof-of-concept pose estimation outputs. We also share mock-up visualisations designed to gather coach feedback. Our aim is to test the feasibility of capturing and combining these data sources and to explore how explainability can turn them into insights that are useful in practice. By making explainability a central design goal from the start, this work contributes to building trustworthy, coach-friendly tools for swimming performance analysis. Overall, this study contributes a novel integration of explainability-driven design with multimodal sensing for swimming. Unlike prior work focusing on algorithmic accuracy, we foreground human interpretability by gathering empirical coach input to inform system development.

## 2. Background and Literature

### 2.1. Multimodal XAI for Sensor-Video Fusion

Multimodal explainability (MXAI) techniques aim to clarify how each data modality (e.g., wearable sensors, video, physiological signals) contributes to a model’s decisions. A common approach is to disentangle the model’s prediction into modality-specific contributions, plus any interaction effects between modalities [3]. For example, DIME (Disentangled Multimodal Explanations) extends LIME to multimodal models by applying local feature attribution on each modality separately and on their fused representation [3]. This yields an importance score per modality, helping quantify, say, how much an IMU sensor stream versus a video stream influenced a swimming stroke classification. Other methods incorporate explainability into the fusion mechanism itself. Attention-based fusion layers can produce learned weights that indicate each modality’s influence on the output [3]. In a sentiment analysis study, researchers computed a numerical “effectiveness metric” from fusion parameters to show how modality interactions (e.g., audio vs. text) contributed to predictions [3]. Likewise, capsule networks have been used to estimate the dynamic contribution of each modality over time [3]. As a simple alternative, ablation analysis can directly score modality importance by measuring performance drop when each modality is withheld [3]. For instance, removing the video input might significantly degrade a model’s accuracy if vision cues were critical, indicating the video’s high importance. Together, these MXAI techniques provide a fusion-level explanation that assigns credit (or blame) to each modality for a given outcome, which is invaluable in a swimming context for understanding whether performance predictions are driven more by biomechanical (video) cues, physiological fatigue signals, or sensor-measured motion patterns.

### 2.2. Hierarchical Prototype Explanations (HIPEs) for Video Actions

Hierarchical interpretability frameworks explain predictions at multiple semantic levels of detail. HIPE (Hierarchical Prototype Explainer) is a recent approach that learns case-based prototypes organised in a hierarchy to interpret video action recognition [1]. Instead of providing a single flat explanation, HIPE aligns with human-like description: for example, an activity might be explained as a water sport, then specifically as swimming, and finally as the butterfly stroke. Gulshad et al. introduce HIPE to connect lower-level class prototypes to higher-level parent classes, leveraging hyperbolic embeddings to naturally encode concept hierarchies [1]. The model identifies prototypical video snippets for fine-grained classes (e.g., a prototype clip of butterfly arm motion) as well as prototypes for broader parent categories (e.g., generic swimming motions). At inference time, the prediction can be explained through a multi-level chain: “the model predicts this is a butterfly stroke because it looks like the prototype for a butterfly, which in turn is a kind of swimming”. This yields a human-understandable rationale like “swimming → butterfly → specific stroke technique.”

The hierarchical structure also helps in cases of uncertainty: if the model isn’t confident at the specific stroke level, it can fallback to explaining the prediction at the parent level (e.g., simply as “water sport”) [1]. An important finding is that incorporating hierarchy can improve the accuracy-interpretability trade-off; HIPE maintained competitive accuracy on the UCF-101 video action dataset while providing richer explanations, outperforming a non-hierarchical prototype model [1]. For sports analytics, this approach is promising—a classification of a swimmer’s activity could be transparently broken down into levels (sport, stroke, sub-technique), aiding coaches in understanding not just what the model predicts, but why at multiple levels of granularity. One implementation challenge is that a predefined taxonomy of actions is required, and the model must learn meaningful prototypes for each category level, which can be complex for subtle skill variations in swimming.

### 2.3. Motion-Relevance Saliency in Video Sequences

For video-based analysis, a key explainability question is: which parts of the motion were most relevant to the prediction? Saliency maps and temporal attention techniques address this by highlighting the frames or spatial regions that the model focused on. A direct extension of image saliency to video is to apply methods like Grad-CAM on each frame or on 3D convolutional blocks. Saha et al. revisited Grad-CAM for action recognition and showed that it can indeed pinpoint motion-relevant regions: for a tennis swing video, Grad-CAM correctly highlighted the moving tennis racket as the salient region leading to the “swing” classification [5]. This indicates the model latched onto the motion of the racket rather than the background pixels. However, a naive frame-by-frame application of saliency ignores temporal context [5]. A significant challenge is capturing temporal saliency—which frames (or sub-sequences) were most crucial [5]. Grad-CAM applied over a sequence must ensure the order of frames is accounted for; if a video’s frames are shuffled or played in reverse, the explanation should reflect changed causality [5]. Research has found that simply treating each frame independently can be misleading, since action understanding depends on the sequence of movements [5].

To tackle this, some explainability methods explicitly use motion information. For example, Zhi et al. propose a motion-guided frame sampling strategy (MGSampler) that inherently emphasises high-motion segments: they compute frame-to-frame differences and assume frames with large changes carry more information about the action [6]. By grouping video frames by motion magnitude, their sampler “explains” its selection by favouring segments where the important action occurs (e.g., the frames during a swimmer’s arm pull underwater, not the gliding between strokes) [6]. In general, temporal saliency maps can be created by attributing importance scores to each frame or time window, often visualised as a timeline highlighting peaks where the model paid attention. Such motion-relevance explanations have been applied in few-shot action recognition and video question-answering to identify key moments of change or interaction. The main implementation hurdle is ensuring spatiotemporal consistency: the highlighted regions must move with the athlete and persist over the critical frames, requiring models to integrate spatial and temporal attention. Despite these challenges, temporal saliency is vital in sports—it could show a coach which part of a dive or turn caused a judging score prediction or which stroke in a lap triggered a fatigue warning, thereby making the AI’s decision process more transparent.

### 2.4. Attention-Based Fusion for Wearable Sensors

When multiple wearable sensors (e.g., IMUs on wrists, trunk, legs) are used to classify athletic movements, attention mechanisms can provide built-in explanations of the data fusion. Instead of treating all sensor inputs equally, an attention-based model learns to assign a weight to each sensor modality or even each time step, indicating its relevance to the current prediction. For example, Mahmud et al. introduced a self-attention network for human activity recognition (HAR) that includes a sensor modality attention layer [7]. This layer produces a set of attention weights (one per sensor stream) that essentially rank the importance of each sensor’s signal in contributing to the recognised activity [7]. In their experiments, these learned weights formed sensor-level attention maps that were “intuitively explainable”, allowing the authors to confirm that, say, for a “walking” activity, the foot-mounted IMU was given higher weight than hand-mounted ones [7]. Earlier, Ma et al. (AttnSense) [8] had combined CNN and GRU layers with both temporal attention and sensor-level attention, enabling the model to focus on salient time periods and sensor channels for multimodal HAR [7,8,9]. Such attention-based fusion not only improved accuracy but also yielded interpretable insights: the model could identify which sensor was most informative for a given action (e.g., a gyroscope on the dominant arm is key for detecting a butterfly stroke, while an accelerometer on the head might be less relevant). A strength of attention weights is that they are directly readable from the trained model for any given input, offering a form of local explanation (per prediction) as well as a global explanation (average attention over many samples) about sensor importance.

Attention maps have been used to build simple visualisations—for instance, a bar chart highlighting that “Sensor 1 (wrist IMU) contributed 50% to the detection of ‘flip turn’, whereas Sensor 2 (ankle IMU) contributed only 20%”. However, it is important to note that raw attention weights should be interpreted with caution. Recent studies have debated whether attention weights truly align with causal importance [3]. In some cases, attention-based explanations can conflict with gradient-based attributions, meaning high weight doesn’t always imply a large effect on the output [3]. Despite this caveat, in practice, attention fusion has proven very useful in wearable sports analytics because it naturally integrates into deep models and provides sensor-level interpretability without separate post-hoc analysis. Implementation challenges include ensuring the attention mechanism doesn’t just attend to sensors with higher noise or magnitude, and validating that what the attention highlights makes sense to human experts (e.g., does the model focus on the IMU that a coach would intuitively consider most important for a given skill?). When designed and validated carefully, attention-based sensor fusion can greatly enhance trust in multimodal sport AI systems by clearly identifying which streams of data are driving each decision.

### 2.5. AI Explainability 360 Toolkit and Taxonomy

To navigate the broad landscape of XAI techniques, researchers at IBM proposed the AI Explainability 360 (AIX360) toolkit, which includes a taxonomy of explainability methods [4,10]. The AIX360 taxonomy is an attempt to systematically organise methods by criteria such as intrinsic vs. post-hoc, global vs. local, and the target stakeholder of the explanation [4]. The premise is that no single explainability method suits all needs—for example, a coach, an athlete, and a data scientist might all require different explanation formats from the same system [4]. AIX360 provides a structured way to pick the right tool for the job. It classifies and implements diverse algorithms covering tabular data, time-series, text, and image modalities in one open-source library [4,10]. This is highly relevant for sports and biomechanics, where datasets are often multimodal (e.g., numerical performance metrics, video, and sensor streams together). Using the AIX360 framework, one can mix and match explainer methods for different components of a system. For instance, in a multimodal injury prediction model with both questionnaire data and motion capture video, the taxonomy might suggest using a feature attribution explainer (like SHAP) for the tabular questionnaire features, and a visual attribution (like Grad-CAM) for the video component [3]. Tjoa et al. [2] demonstrated a combined approach in a medical context: they applied SHAP to handle patient metadata (tabular features) while using Grad-CAM on imaging data, creating a joint explanation for a skin lesion diagnosis model [2].

A similar approach could be taken in a swimming analytics system: SHAP values could explain sensor-derived features (e.g., highlighting that “muscle $O_{2}$ saturation drop was a strong contributor to the fatigue flag”), while visual saliency overlays explain video-based features (e.g., highlighting the moment a swimmer’s elbow dropped, causing a technique fault). The AIX360 toolkit not only provides these algorithms but also metrics to evaluate explanations and tutorials to guide practitioners [4]. By following a taxonomy-driven approach, developers can ensure that for each modality and user type in a sports system, an appropriate explainability method is chosen. In summary, AIX360 encourages a systematic, multi-method explainability strategy: rather than relying on a single “one-size-fits-all” explainer, it promotes orchestrating several techniques (rules, feature attributions, prototypes, saliency maps, etc.) so that every aspect of a complex model (and every stakeholder) is covered by an understandable explanation [4]. This philosophy aligns well with the needs of high-performance sports environments, where coaches might demand human-interpretable rules and summaries, whereas sports scientists might appreciate quantitative feature impact charts—all of which can be integrated under a unified XAI framework.

### 2.6. Traditional XAI Methods in a Sports Context

Many traditional explainability methods from machine learning can be adapted to the sports analytics domain, often by customising them to sensor or video data. For sensor-based models, common model-agnostic tools like SHAP and LIME are popular for explaining predictions. SHAP assigns each feature a contribution value for a particular prediction, derived from game-theoretic principles. In athlete monitoring research, SHAP has been used to identify influential factors in performance outcomes—for example, one study on predicting young athletes’ fitness found that SHAP analysis could clearly rank the importance of features such as training load, age, and $VO_{2}$ max in determining fitness levels [11]. This gave coaches insight into why the model might rate one athlete’s fitness higher than another (e.g., a higher training load had a positive SHAP value for the fitness score). LIME can likewise be applied by perturbing sensor inputs (like slightly altering stride length or stroke count features) to see how the prediction changes; however, LIME’s use in multimodal settings can be tricky, which is why methods like DIME were developed to handle each modality separately [3].

Aside from these post-hoc methods, simpler feature importance metrics are often reported when using tree-based models or linear models on sports data. For instance, a random forest that predicts swim race time from biomechanical metrics can output an importance value for each input (e.g., showing that stroke rate is the top contributor, followed by turn time, etc.). Partial dependence plots (PDPs) are another classic tool: they chart the model’s predicted outcome as a function of one feature, averaging out others. In a swim analysis context, a PDP could reveal a non-linear relationship, such as “beyond 50 strokes per minute, further increases in stroke rate yield diminishing improvements in speed,” which is valuable for training decisions. One recent study compared different neural network models on wearable HAR data and applied Layer-wise Relevance Propagation (LRP) to interpret them; the LRP results showed, for example, that gyroscope Y-axis data was consistently the most informative sensor input for distinguishing activities, whereas certain other channels contributed little [12]. This kind of insight—that “rotation about the Y-axis is key for this movement”—aligns with domain knowledge and validates the model’s focus. Furthermore, in sports settings, there is still a role for transparent, rule-based explanations. Traditional expert systems and simple threshold rules have long been used by coaches, precisely because their logic is easy to follow. For instance, a rule-based algorithm might flag “possible fatigue” if a swimmer’s stroke rate drops by >10% while heart rate is high. Such approaches are highly interpretable and deterministic, as each decision can be traced to a clear rule [13]. Despite the limitations of handling complex patterns, these rule-based methods provide strong transparency and have been integrated even in modern hybrid systems (sometimes alongside machine learning) to ensure certain outputs remain understandable [13]. In summary, for sensor modalities, the practitioner has a spectrum of XAI options—from advanced methods like SHAP that quantify each feature’s contribution, to straightforward if-then rules or feature rankings—and combining these can yield both rigorous and coach-friendly explanations (e.g., “SHAP says lap time is the top factor, and indeed whenever lap time > X, our rule triggers an alert”).

For video and pose data, traditional explainability often takes the form of visualisations. Saliency maps (heatmaps overlayed on video frames) derived from methods like Grad-CAM, RISE, or guided backpropagation can show which body regions or objects in the scene influence the model’s decision [3]. In action recognition tasks, these saliency techniques have been extended to highlight not only spatial areas but also correspond to specific frames [5], effectively indicating “when” and “where” the model paid attention. In a swimming video, a saliency overlay might light up the swimmer’s kicking legs during a freestyle sprint if the kick technique drives the classification of the stroke intensity. Another approach is to leverage the outputs of pose estimation directly: since pose models produce interpretable joint coordinates, skeletal stick figures can be drawn on the video to represent the swimmer’s motion. Many studies that use pose data for feedback simply present the reprojected skeleton or keypoint trajectories on the video. While this is technically a form of data visualisation rather than an explanation of a black-box model, it serves an explainability purpose by linking the analysis to something the coach can see—for example, highlighting an asymmetry in left vs. right-hand trajectories through the water.

Additionally, researchers have proposed overlaying vector fields or force arrows on athlete videos to visualise computed forces or motion directions. In swimming, this could mean displaying arrows along the swimmer’s hand path indicating propulsive force magnitude and direction, thus explaining why a particular stroke was labelled inefficient (e.g., the force vectors show a lot of lateral slippage instead of forward thrust). Attention-based methods also translate to visual explanations: if a transformer model outputs an attention map over video frames, this can be depicted as frame-wise highlighting or a timeline curve showing peaks—analogous to a coach’s eye focusing on the critical moment of a turn. The common thread is that for visual data, explanations tend to be visual. This aligns with coach feedback: heatmaps on a replay, annotated videos, and overlayed analytics are often more intuitively understood than numbers or text [3]. The main challenge is ensuring these visual explanations truly correlate with model reasoning (avoiding random or misleading highlights) and that they can be generated efficiently (ideally in real-time for feedback during training). Nonetheless, adapting saliency and interpretability tools to sports videos is a highly active area, bringing computer vision explainability techniques into practical use on the pool deck.

### 2.7. Comparative Analysis and Implementation Challenges

Each of the above frameworks offers a different balance of insight, complexity, and usability, and combining them effectively in a sports analytics system requires careful consideration. A fundamental trade-off is between intrinsic interpretability and model accuracy [1,2]. Techniques like prototype models (HIPE) or rule-based systems bake interpretability into their structure, but they may sacrifice some predictive performance or require simplified model architectures. Conversely, post-hoc explainer methods (SHAP, LIME, saliency maps) can be applied to any high-performance model, but their reliability and fidelity become concerns. Researchers have noted that adding interpretability often comes at a cost—either in accuracy or in additional computation—though clever design can mitigate this [1]. For instance, HIPE’s use of hyperbolic embeddings maintained accuracy while providing multi-level explanations, partially resolving the typical accuracy-explainability tension [1].

Another challenge is evaluating and validating explanations, especially in multimodal settings. Unlike predictive accuracy, there is no universally agreed metric for explanation quality. It is difficult to say whether an attention heatmap or a SHAP summary is a “good” explanation without reference to human judgment. In fact, different XAI methods can disagree—an attention-based explanation might highlight different features than a gradient-based explanation for the same prediction [3]. This lack of consistency raises questions: which explanation should we trust? Efforts are underway to develop benchmarks and ground-truth datasets for explainability, but currently, the evaluation of XAI in sports is often subjective. Experts might need to verify that what the model deems important (e.g., a certain kinematic pattern) aligns with coaching knowledge. The absence of ground-truth visual explanations (e.g., labelled “important” video segments from coaches) and standardised evaluation protocols makes comparative assessment of these techniques difficult [3]. This is an active research area, and improving it will help identify which XAI methods truly aid decision-making in practice.

A related issue is the faithfulness vs. interpretability dilemma: Some explanations are simple and appealing (like “Sensor X is most important”), but they risk oversimplifying the model’s logic. There is a danger of coaches drawing false confidence from an explanation that only tells part of the story. For example, a partial dependence plot might show a clear trend of “more distance per stroke is better,” but the model might also be internally using other variables and interactions that the PDP doesn’t convey. Thus, combining multiple explanation types can be beneficial, providing both a high-level intuitive rule and a more detailed feature attribution, yet this also means more information for the user to process.

Real-time constraints pose another implementation challenge in sports environments. Unlike offline analysis, a coaching feedback system may need to generate explanations on the fly (e.g., right after a swimmer finishes a lap). Techniques like SHAP and LIME can be computationally intensive, as they require many model evaluations per explanation [3]. Approximations or simplified surrogate models might be needed to deliver explanations quickly. On the other hand, attention weights or prototypes are obtained directly from the model’s forward pass and can be more real-time friendly. System designers must choose explainer methods that fit within time and resource budgets, possibly using pre-computed explanations or caching to speed things up.

Perhaps the most crucial factor is human interpretability and user acceptance. The end goal of XAI in sports is not just to satisfy academic interest, but to genuinely assist coaches and athletes. Explanations should be presented in forms that stakeholders find meaningful, which may vary. A coach with a limited data science background might prefer a simple visual or a natural-language summary (e.g., “The AI flags a drop in elbow height as the reason for speed loss in your last 50 m”). A sports scientist or biomechanist might appreciate a more quantitative explanation (e.g., a chart of feature importances or a comparison to prototypical elite performance data). Therefore, tailoring the explanation to the end-user’s expertise and cognitive needs is essential [3]. Research underscores that one-size-fits-all explanations often fail [4]—non-experts can be overwhelmed or misled by technical details, whereas domain experts may require more depth and precision [3]. Achieving the right personalisation means involving users in the design (co-operative design of dashboards, user studies on interpretability) and possibly offering multi-tiered explanations (an overview first, with the ability to drill down for more detail).

In comparing the techniques, Model-specific vs. agnostic methods are a key distinction. Prototype learning, attention mechanisms, and hierarchical models require designing (or at least retraining) the model in a special way—they offer built-in explanations which are often more direct and stable (since they reflect the model’s actual components), but at the cost of flexibility. In contrast, agnostic methods like SHAP, LIME, or LRP can explain any model after the fact; they are very flexible and can be swapped out as needed, yet one must trust that these approximations accurately reflect the true reasoning of the model [3]. Ensemble or hybrid strategies (as advocated by AIX360) try to get the best of both worlds by using intrinsic interpretability where feasible and supplementing with post-hoc explanations elsewhere.

Lastly, integration and visualisation of explanations in a user interface is a non-trivial practical challenge. A multimodal system might generate several pieces of explanation: heatmaps on video, graphs for sensor feature importance, textual descriptions, etc. Presenting this coherently in a “coach-facing dashboard” without overwhelming the user is difficult [14]. Early prototypes in the literature have used traffic-light indicators, simple icons, or concise textual hints on dashboards to convey model findings. The design should avoid cognitive overload and focus on actionable insight: the explanation should answer a coach’s implicit questions like “what should I look at in this performance?” or “why did the AI flag this segment?”. Achieving this requires interdisciplinary effort—combining the robust algorithms from XAI research with HCI (Human-Computer Interaction) principles and domain knowledge in sports.

**Figure 1:**
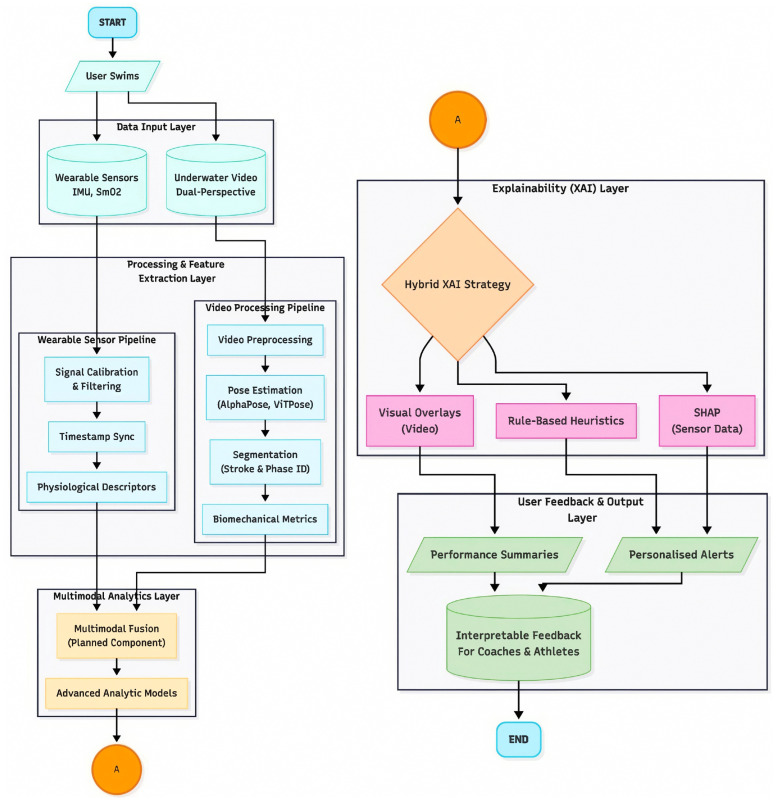
Conceptual architecture of the proposed SWIM-360 system. The workflow begins with data collection from wearable sensors (IMU, SmO2) and dual-perspective underwater video. The processing pipeline extracts physiological and biomechanical descriptors, aligns sensor and video data, and feeds them into a planned multimodal analytics layer. The Explainability (XAI) Layer applies a hybrid strategy, combining visual overlays, SHAP-based feature attributions, and rule-based heuristics to generate interpretable feedback for coaches and athletes through performance summaries and personalised alerts.

Figure 1 illustrates the conceptual architecture of the SWIM-360 system, which integrates multimodal sensing, explainability mechanisms, and user-centred feedback. The process begins with data capture from wearable inertial and near-infrared spectroscopy (NIRS) sensors together with dual-perspective underwater video. These data streams pass through calibration, synchronisation, and feature-extraction pipelines before being combined within a planned multimodal analytics layer. A dedicated explainability layer implements a hybrid XAI strategy that merges SHAP-based feature attribution for sensor data with visual overlays and rule-based heuristics for video insights. The resulting feedback is aggregated into a coach-facing interface that provides interpretable performance summaries and personalised alerts.

In summary, the reviewed literature highlights persistent gaps in multimodal explainability for sports analytics, including (1) limited integration of sensor and video modalities within a unified explainability framework, (2) the absence of domain-specific evaluation protocols for interpretability, and (3) minimal engagement of end-users, such as coaches, in the design and validation of AI systems.

To address these gaps, the SWIM-360 project applies explainability methods to real-world swimming analysis through an iterative, user-centred approach. Drawing on frameworks such as HIPE, attention-based fusion, and the AIX360 taxonomy, SWIM-360 translates abstract multimodal explainability principles into design requirements that are tested and refined in collaboration with coaches. By embedding explainability and usability considerations at every development stage, the project bridges the methodological divide between theoretical research and practical application in aquatic sport environments.

Feedback from pilot testing has guided refinements in multimodal data fusion, visual feedback, and usability design, ensuring that interpretability aligns with coaching practice and training objectives. This iterative validation process addresses the lack of participatory design in prior studies and establishes a foundation for domain-specific evaluation of explainable outputs.

Collectively, these developments show how SWIM-360 operationalises multimodal explainability as both a technical and human-centred objective. Rather than benchmarking isolated models, the project focuses on transforming explainable AI methods into actionable, interpretable feedback mechanisms tailored to the needs of coaches. This approach advances the field by demonstrating how user-informed design can turn multimodal explainability research into transparent and trustworthy decision-support systems for performance analysis.

## 3. Methodology

### 3.1. Overall Review

The methodological approach adopted in this study followed an explainability-driven and human-centred design process, integrating principles from multimodal sensing, XAI, and participatory system design. The purpose was not only to test technical feasibility but to understand how coaches interpret, trust, and make use of multimodal feedback in swimming. To achieve this, a structured questionnaire was designed to capture professional coaches’ expectations and preferences for explainable feedback, forming the empirical foundation for subsequent dashboard and model-development phases. Given the exploratory scope and small participant pool (n = 8), the study was designed to collect qualitative insights rather than produce statistically generalisable results. Participants were recruited through professional swimming coaching networks in Malta and the EU, with inclusion criteria requiring at least two years of coaching experience.

The questionnaire was conceived in direct response to the theoretical and technical insights reviewed in Section 2. As discussed there, the literature on explainable multimodal AI emphasises that successful implementation depends on aligning algorithmic transparency with the informational needs of human stakeholders [3,4]. Frameworks such as AIX360 [4] underline that no single explanation method suits all audiences; rather, the form and depth of explanation must adapt to the user’s role and expertise. Coaches, who ultimately interpret sensor outputs in context, therefore became the focal participants in the early design phase of SWIM-360. The questionnaire served as a mechanism for eliciting these user requirements, so that later technical decisions could be informed by an evidence-based understanding of what kinds of explanations are meaningful in real coaching environments.

The design process began with a mapping of conceptual dimensions identified in Section 2, interpretability, modality importance, visual saliency, temporal explainability, and stakeholder relevance, to the structure of the questionnaire. The resulting questionnaire consisted of four sections (General Information, Visualisation Preferences, Practical Use, and Technique and Biomechanical Feedback), each targeting a distinct aspect of explainable system design. The format combined short multiple-choice and ranking items with open-ended responses to balance quantifiable comparison and qualitative insight. All items were developed from first principles derived from XAI theory and multimodal sensing research, ensuring that each question had a methodological justification rather than being merely descriptive.

The opening section established contextual variables relevant to explainability. Questions about the swimmers’ level and the coach’s country of practice acknowledged that information needs vary across competitive levels and cultural settings, echoing prior observations that explainability is context-dependent rather than universal [3]. Items on the number of swimmers per session helped define system scalability, reflecting parallels with multimodal fusion models where attention mechanisms distribute importance across multiple input streams [7,8]. Respondents were also asked to indicate which feedback categories, techniques, physiology, performance, or injury prevention were most useful. These domains correspond to the sensor modalities explored earlier in the paper (IMU, NIRS, and video), linking the human-facing survey directly to the technical architecture of the SWIM-360 system. The first section concluded with a Likert-scale item evaluating the perceived importance of explainability itself, derived from AIX360’s emphasis on stakeholder-specific interpretability needs [4]. This item served as a benchmark for assessing how strongly transparency is valued relative to raw performance accuracy in real coaching contexts.

The second section examined preferences for visual explanations, integrating lessons from research on perceptive and multimodal interpretability [2,5]. Since visual overlays such as saliency maps and motion highlights have been shown to enhance understanding in action-recognition models [5,6], respondents were asked to select their preferred visualisation type, video replay with highlights, colour-coded feedback, trend graphs, or side-by-side comparisons, and to rate how helpful each would be in everyday coaching. These questions directly operationalised the principle that explanations should exploit the modality through which the underlying data are experienced: in this case, the predominantly visual nature of swimming technique. By contrasting alternative display types, the survey also allowed investigation of how different representational forms support or hinder interpretability across varying levels of analytical detail.

The third section focused on practical use and temporal context, drawing from the literature on human-centred explainability and system usability. Questions on preferred data format (numerical values versus interpreted statements) explored the balance between raw data transparency and semantic summarisation, reflecting the ongoing discussion between intrinsic and post-hoc explanations [3]. Items about information density and timing of feedback (real-time versus post-session) addressed cognitive and operational constraints. Real-time explanation poses known computational challenges; SHAP and LIME, for example, require repeated model evaluation, whereas attention-based mechanisms offer faster, built-in interpretability [7,9]. Capturing user priorities here helped determine which technical strategies would be viable for future dashboard deployment. Finally, questions on device type and usage frequency grounded the design in real coaching environments, ensuring that the proposed system adapts not only to users’ expertise but also to their working context, whether poolside on a tablet or during detailed desktop analysis.

The final section of the questionnaire concentrated on technique-specific and biomechanical explainability, linking directly to the sensor analysis components of SWIM-360. Respondents rated the importance of metrics such as body alignment, kick symmetry, breathing timing, and turn efficiency, features corresponding to those emphasised in prototype-based and attention-based models for HAR [1,8,12]. These items translated theoretical variables into domain-specific terms familiar to coaches, ensuring conceptual continuity between algorithmic interpretability and biomechanical reasoning. The open questions that followed invited participants to describe the key elements they would most want monitored, the challenges of performance analysis without technology, and the attributes that would make the SWIM-360 dashboard most useful. These qualitative prompts were guided by participatory design principles and the co-creation ethos advocated in explainable AI research [3]. They sought to uncover coaches’ implicit mental models, the heuristics and visual cues they already rely on, so that future system explanations could align with established professional reasoning rather than introducing alien concepts.

The questionnaire underwent internal pilot testing with researchers familiar with aquatic performance analysis. Revisions improved clarity and reduced redundancy between overlapping items, while maintaining a balance between brevity and conceptual coverage. A mixture of scaled and open responses ensured both statistical and interpretive richness. Ethical procedures were integrated throughout the design. Open-ended responses were analysed thematically by two independent reviewers using an inductive coding approach. Agreement on key themes exceeded 85%, ensuring consistency of interpretation. No personal identifiers were collected; all participation was voluntary. The consent statement at the beginning of the questionnaire functioned as both an ethical safeguard and a methodological parallel to explainability principles, explicitly informing participants how their data would be used, mirroring the system’s eventual obligation to explain its outputs to end-users. For a more detailed view on each question’s format and decision, refer to Appendix A. The study was approved by the University of Malta Research Ethics Committee (Ref: UM-REC-2025-03)

### 3.2. Preliminary Results

**Figure 2:**
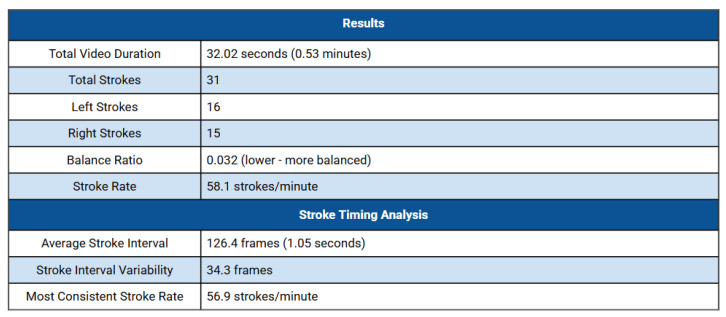
Summary of stroke-related metrics extracted from the video-based analysis. The table reports total stroke count, left–right stroke distribution, balance ratio, and overall stroke rate, followed by stroke-timing indicators derived from frame-level detections. These measures provide an initial quantitative overview of temporal stroke characteristics across the analysed segment.

The preliminary multimodal measurements confirm that the system can reliably acquire and synchronise heterogeneous data streams under real swimming conditions. The video-based pose-estimation component yielded stable joint detections throughout the underwater sequence, enabling the identification of stroke phases as seen in Figure 2, and key temporal events. These outputs support lap-to-lap comparisons of timing characteristics and stroke counts, providing a consistent basis for analysing stroke patterns as well as their temporal evolution. Although the video-processing module is still being tuned, the current results indicate that the overall approach is technically feasible and promising for future large-scale deployment.

**Figure 3:**
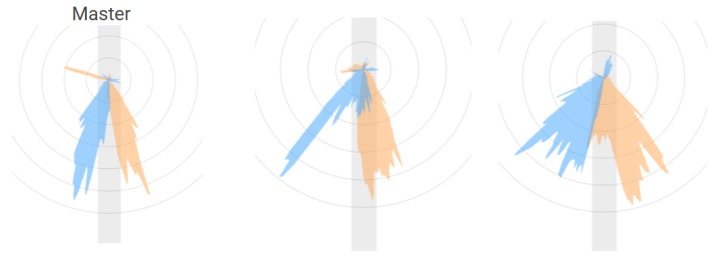
Comparison of force-field patterns derived from EO hand-mounted sensors for swimmers of different proficiency levels during freestyle. The reference “Master” swimmer (**left**) displays a compact and symmetric distribution of propulsive force vectors, whereas less experienced swimmers (**middle**,**right**) exhibit broader and more asymmetric patterns, indicating variations in hand trajectory, force orientation, and overall stroke consistency.

The EO SwimBETTER force sensors (eo, Sydney, Australia) yielded detailed biomechanical profiles of hand-applied propulsion as seen in Figure 3. Early recordings revealed clear left–right disparities in both force magnitude and directional distribution. Such asymmetries may arise from imperfect stroke strategy, habitual compensatory patterns, or stroke-side breathing in freestyle, which naturally introduces rotational and timing differences. The resulting force-field characteristics appear sufficiently sensitive to detect subtle deviations in technique that might not be captured through visual inspection alone. Observing the persistence or lap-specific variation of these asymmetries may also offer valuable context for understanding mechanical consistency and overall stroke effectiveness.

**Figure 4:**
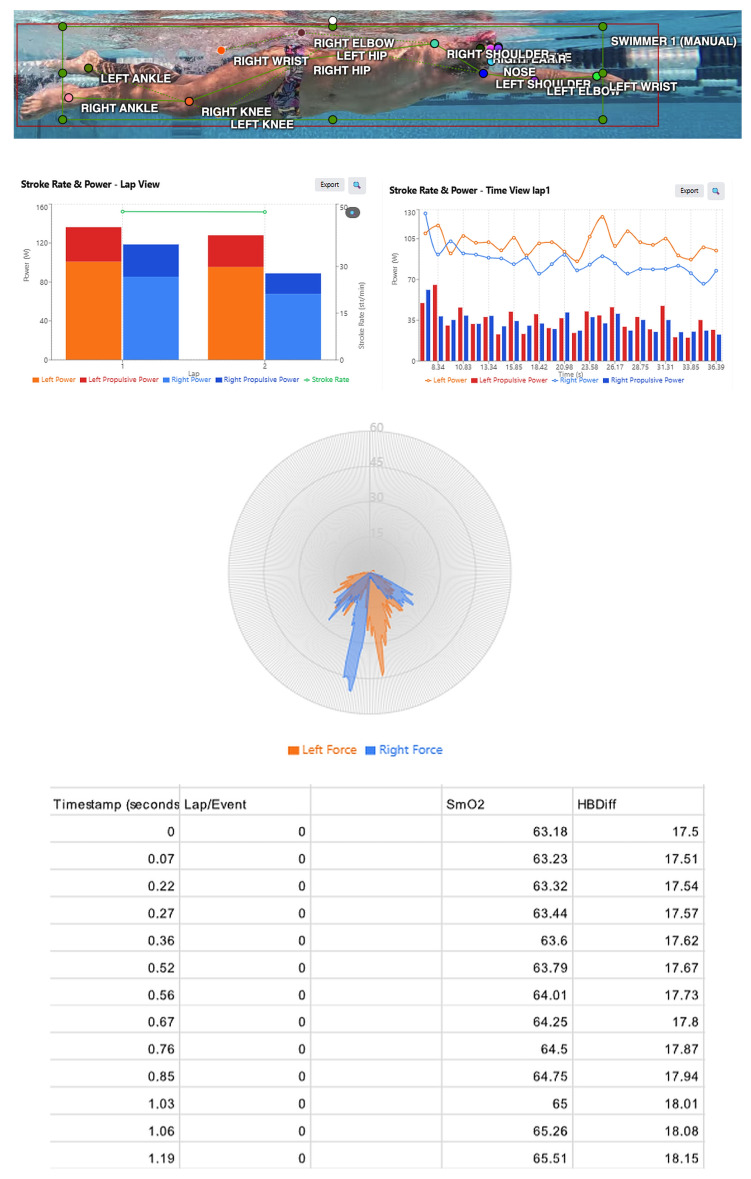
Preliminary multimodal outputs combining video-based pose estimation, EO SwimBETTER (eo, Sydney, Australia) force measurements, and Train.Red NIRS data. The top panel illustrates detected joint keypoints during an underwater stroke cycle. The middle plots display stroke-specific force and power characteristics, including left–right propulsive contributions and force-vector distributions. The lower table presents corresponding SmO2 and HbDiff values sampled throughout the lap, providing a concurrent physiological profile of muscular oxygenation and haemodynamic response.

As seen in Figure 4, the TrainRed (Train. Red B.V., Gelderland, The Netherlands) NIRS measurements provided an additional physiological dimension by capturing the swimmer’s muscle oxygenation dynamics. The SmO2 and HbDiff trends reflect the athlete’s initial readiness at the start of the session and the subsequent evolution of local muscular demand during repeated stroke cycles. When interpreted alongside the mechanical and temporal indicators, these physiological profiles offer a broader understanding of the swimmer’s condition and the factors that may influence variations in propulsive effectiveness. This alignment across modalities illustrates the potential of integrating metabolic-state information with biomechanical and temporal markers within a unified monitoring framework.

Taken together, these preliminary findings show that video-derived kinematics, hand-based propulsive force measurements and physiological signals can be examined within a unified multimodal system. Viewed together, the three modalities provide a clearer picture of stroke execution by relating temporal characteristics, such as stroke counts, phase timing and rhythm, to the measured propulsive forces and the observed physiological responses. This combined perspective helps describe how mechanical effectiveness, coordination and physiological state develop during the session, offering insights that are not available when each data stream is considered on its own.

## 4. Results

A total of eight responses were collected from practising swimming coaches who completed the SWIM-360 questionnaire. The purpose of this stage was exploratory: to capture qualitative insights into coaches’ priorities, preferences, and perceived challenges regarding explainable multimodal feedback, rather than to achieve quantitative generalisation. All respondents provided informed consent and completed the full survey.

Although most coaches worked primarily with age-group swimmers, several also coached national-level and masters athletes, reflecting a range of competitive contexts. The typical number of swimmers monitored per session varied widely, from 1–4 individuals in small-group technical training to 11–20 in larger team environments. This spread indicates that feedback tools must accommodate both detailed, athlete-specific visualisation and efficient overviews suitable for group sessions.

Across respondents, there was near-unanimous agreement that the most useful forms of system feedback would include stroke technique, physical condition, and performance metrics. Several also emphasised injury prevention as an important though secondary concern. When ranking key coaching priorities, stroke efficiency and fatigue or recovery consistently appeared at the top, followed by race performance, with injury prevention often rated as somewhat less critical in day-to-day training contexts.

The perceived importance of explainability was high across all submissions. On the five-point scale, most coaches rated it either 4 or 5, indicating that they value systems capable of clarifying why particular results or alerts occur. The same tendency appeared when the question was repeated in the context of visual outputs, confirming that interpretability is not only valued conceptually but also expected to be visible in practical feedback displays.

When asked about preferred visualisation formats, the majority selected video replay with highlights, often accompanied by comments describing how athletes “can’t see themselves swim” and how visual playback aids correction. Regarding overall interface preferences, seven of the eight coaches selected a customisable dashboard view as the screen they would most likely use daily. This suggests a strong demand for flexibility in how information is displayed, allowing users to tailor the interface to the focus of each training session. Only one respondent preferred a fixed “stroke technique view,” indicating that while detailed biomechanical data are valued, they should be presented within a configurable structure.

When rating the usefulness of individual visual features, video highlights and colour-coded feedback received the highest ratings overall, typically 4–5 on a five-point scale. Graphs and trend lines were seen as helpful for tracking progress over time, but not necessarily as primary tools during live coaching. Side-by-side comparisons were considered particularly valuable for illustrating technique differences, especially in age-group and masters coaching, where reference to elite form can clarify corrective goals.

Half of the coaches preferred a side-by-side display of numerical data and system-generated interpretations (e.g., stroke rate alongside “below optimal range”). A smaller group preferred to see only the numerical outputs, while just one coach was satisfied with viewing only the interpreted results. This pattern suggests that presenting both forms of data together best supports understanding and trust in the system’s feedback.

When asked about information density, nearly all respondents opted for key highlights with the option for details, underscoring a shared concern about information overload. This pattern indicates that an effective interface should prioritise clarity and brevity while still allowing expert users to explore deeper analytics when needed.

In terms of the timing of feedback, several respondents described real-time information as essential, while others considered post-session review sufficient. Those coaching smaller groups were more inclined to request immediate feedback, often citing the desire to “correct mistakes exactly when they happen,” whereas those managing larger squads prioritised efficient summary reports.

Device preferences reflected the mobility of the poolside environment. Tablets and smartphones were the most frequently selected platforms, with some also mentioning laptop use for detailed analysis and large screens for group feedback. This distribution reinforces the need for a responsive design adaptable to multiple contexts of use. Regarding frequency, coaches indicated they would access the dashboard anywhere from every session to a few times per week, showing that the system must be both reliable for daily operation and lightweight enough to integrate smoothly into existing routines.

In the section on technique and biomechanical feedback, all metrics were rated as highly important, with mean scores typically between 4 and 5 on the importance scale. The most valued parameters across strokes were arm pull depth and efficiency, body alignment and rotation, and start, turn, and push-off efficiency. Breathing timing and kick symmetry were also rated positively but were occasionally ranked slightly lower, suggesting that these may be more stroke-specific priorities.

Open-ended responses offered further qualitative insight. When asked to identify three key elements to monitor, coaches most often mentioned stroke rate, body position or alignment, underwater efficiency, and recovery metrics. One respondent highlighted “heart rate, strokes per minute, and comparisons with previous sessions,” reflecting a desire to combine biomechanical and physiological feedback.

When describing the biggest challenges faced without technology, several themes emerged. Coaches frequently noted the difficulty of observing all swimmers simultaneously, the impossibility of athletes seeing themselves swim, and the risk of forgetting to provide timely corrections when working with larger groups. The absence of objective, quantitative data was also cited as a limitation, particularly concerning recovery and training load management.

Lastly, in response to what would make the SWIM-360 system most useful, coaches converged on the need for ease of use, mobile accessibility, and integration of real-time and comparative feedback. Some emphasised the importance of being able to share information directly with athletes to support self-reflection, while others requested features that could “make the efficiency improvement process shorter” by automatically highlighting performance deviations. For a more detailed view on all responses, refer to Appendix B.

While the sample size was limited (n = 8), the consistency across responses indicates reliable trends in coach preferences. The exploratory design is therefore appropriate for defining system requirements rather than statistical generalisation. A summary of the results is available in Table 1 below:

## 5. Discussion

The results of the coach questionnaire provide early empirical evidence for how explainability can be meaningfully integrated into multimodal sensing systems for swimming. It is important to note that these findings are based on feedback from a small sample of eight professional coaches. While this number limits statistical generalisation, the qualitative design was intentionally chosen to capture exploratory insights into user expectations and interpretability requirements at an early stage of development. Consequently, the findings should be viewed as formative rather than confirmatory. Nonetheless, the consistency of qualitative themes across submissions reinforces key points identified in the literature on MXAI, attention-based fusion, and user-centred interpretability. Together, these insights form a foundation for translating technical XAI concepts into practical, coach-facing design principles.

An additional methodological limitation concerns the questionnaire design. Questions Q6 and Q7 in Appendix A were phrased in a nearly identical way (“How important is it that the system explains why a certain result or alert was generated?” and “How important is it that the system explains why a result or alert appears?”). This redundancy may have led participants to interpret the items as referring to the same construct, potentially reinforcing or inflating the apparent importance of explainability in the results. However, this overlap does not affect the qualitative insights derived from open-ended responses.

Despite these limitations, the responses reveal a clear pattern in how coaches value transparency and interpretability. Almost all participants rated explainability as “important” or “very important,” both in general terms and in visual contexts. This aligns closely with the premise of the AIX360 framework [4], which argues that transparency should be calibrated to stakeholder needs. For the SWIM-360 project, the end-user is neither a data scientist nor an athlete, but a coach operating in a time-constrained, information-rich environment. The demand for explanations, why a particular result or alert appeared, demonstrates that black-box outputs are insufficient in applied sport contexts. As AIX360 highlights, explainability is not merely a model property but a form of communication between algorithm and user, and these findings affirm that coaches expect such dialogue to be part of the system interface.

The strong preference for visual interpretability provides further validation of the multimodal XAI approaches reviewed in Section 2. Video replay with highlights was by far the most popular feedback modality, followed by colour-coded overlays and side-by-side comparisons. These preferences correspond directly with techniques such as Grad-CAM saliency mapping [5] and motion-guided frame selection [6], which emphasise where and when the model attends within a sequence. Coaches’ comments that “athletes can’t see themselves swim” and “video analysis helps correct mistakes” indicate that visual explanations are not only comprehensible but functionally embedded in existing coaching workflows. Such visualisations effectively serve as intrinsic explainers, bridging the gap between algorithmic reasoning and the perceptual experience of movement.

Interestingly, several respondents requested comparative or reference-based views (e.g., “side-by-side with elite swimmers”), echoing the structure of HIPE [1]. HIPE models organise interpretations at multiple semantic levels, such as “swimming” → “freestyle” → “specific stroke technique,” providing a human-like hierarchy of understanding. Translating this idea into SWIM-360 would allow explanations that operate on multiple conceptual scales, for instance, identifying an overall deviation in stroke class while also pinpointing the precise biomechanical feature causing inefficiency. The coaches’ emphasis on contextual benchmarking demonstrates a preference for relational explanations, where meaning arises through comparison rather than absolute metrics.

The finding that most participants favoured customisable dashboard views reinforces the argument that explainability must be adaptive to user goals [3]. In multimodal XAI, adaptivity often refers to the fusion stage, where attention mechanisms or disentangling explainers (like DIME) adjust modality weights dynamically, but the same principle applies at the human-computer interface. A customisable layout allows the user to surface the modality or explanation type most relevant at a given moment, reflecting a practical form of interactive interpretability. The strong demand for such flexibility parallels the attention-based fusion frameworks proposed for wearable sensors [7,8], where the system learns to focus on the most informative inputs. Here, the coach plays an analogous role: directing attention across feedback modalities depending on training objectives.

Equally significant is the balance between raw and interpreted data preferred by participants. Half of the coaches requested both numerical and semantic outputs side by side, illustrating the dual requirement of transparency and abstraction. This resonates with the multimodal taxonomy proposed by Rodis et al. [3], in which local quantitative explanations (e.g., SHAP values for sensor features) are complemented by higher-level verbal or visual summaries. For SWIM-360, this finding suggests that explanations should not replace raw performance data but contextualise them, ensuring that interpretability does not obscure domain-relevant detail.

Responses concerning timing and cognitive load mirror broader tensions discussed in explainability research, specifically, the trade-off between real-time utility and explanation richness. Coaches working with small groups tended to prioritise immediate feedback, whereas those managing larger teams accepted post-session review. This divide parallels the computational trade-offs identified in multimodal XAI systems [3], where model-agnostic methods like SHAP or LIME can be too slow for live deployment, and attention-based mechanisms or intrinsic prototypes offer faster, if less detailed, interpretability. These qualitative insights thus provide design guidance for later implementation: real-time views may rely on lightweight, built-in attention or rule-based explanations, while post-session analysis can incorporate more computationally intensive model-agnostic methods.

The consistent selection of mobile devices (particularly tablets and smartphones) underscores the situational constraints of aquatic environments. From an interpretability standpoint, this highlights the importance of visual economy: explanations must convey meaning in compact visual forms without requiring extended analysis time. As noted in medical and sports XAI applications, interpretability is partly a function of interface bandwidth, the amount of attention and time a user can dedicate to understanding a result [2]. Designing for limited display space and rapid comprehension, therefore, becomes a practical extension of the explainability problem.

The biomechanical feedback section of the questionnaire provides a direct bridge between sensor-level interpretability and domain-level insight. Coaches rated arm-pull efficiency, body alignment, and start or turn performance as the most important indicators across all strokes. These preferences closely align with findings from attention-based HAR models such as AttnSense [8] and sensor-level attribution studies [12], which identified gyroscope and accelerometer data related to limb rotation and propulsion as key explanatory features. This overlap suggests that the model’s internal logic, if designed to highlight these variables, would naturally correspond to the parameters coaches already deem most meaningful. Such alignment between human and algorithmic reasoning is central to trustworthy explainability, indicating that SWIM-360’s future sensor models should emphasise interpretive coherence over novelty.

As outlined in Figure 1, the forthcoming SWIM-360 prototype will operationalise the multimodal fusion and hybrid explainability pipeline to deliver post-training visuals and interpretable insights to coaches.

Open-ended responses further reveal the epistemic function of explainability for coaches. Participants repeatedly mentioned the difficulty of observing all swimmers, the challenge of delivering timely corrections, and the need for objective recovery metrics. These statements illustrate that explainability is not merely about validating AI predictions but about extending human perception, making invisible aspects of performance (such as underwater motion or physiological recovery) visible and actionable. This interpretation resonates with the broader definition of explainable systems as cognitive extensions of human expertise rather than post-hoc justification tools [3].

The emphasis on ease of use and integrated, shareable feedback reflects the socio-technical dimension of explainability. Trust and adoption depend not only on what the model explains but also on how seamlessly the explanation fits within existing coaching practices [14]. As highlighted by both AIX360 and Rodis et al. [3], successful explainability requires careful orchestration between algorithmic, visual, and interactive components. In SWIM-360, this orchestration will involve linking attention-derived sensor weights, visual saliency overlays, and contextual natural-language summaries within a unified, coach-facing interface.

In the next development phase, SWIM-360 will integrate IMU, NIRS, and video data streams using attention-based fusion. Explainability outputs (e.g., SHAP values, attention weights, and visual saliency maps) will be linked to a coach-facing dashboard derived from the preferences reported here. A preliminary prototype will be evaluated for interpretability and usability.

## 6. Conclusions

This paper presented early findings from the SWIM-360 project, an initiative exploring how explainable multimodal sensing can enhance swimming performance analysis. Building on theoretical frameworks of explainable artificial intelligence, the study designed and deployed a qualitative questionnaire to capture coaches’ perspectives on feedback, visualisation, and interpretability.

The results indicate that coaches place strong value on transparency and contextual understanding, preferring visual explanations such as video highlights and colour-coded overlays that clarify why a result occurs rather than simply presenting data. They also emphasise the importance of customisable and adaptive interfaces, real-time feedback where feasible, and integration of both biomechanical and physiological indicators. These preferences align closely with the multimodal explainability methods discussed in earlier sections, supporting the relevance of attention-based fusion, hierarchical prototypes, and taxonomy-guided design approaches for future system development.

These findings underline that explainability is a foundational usability feature, not a secondary add-on, for AI-driven coaching systems. The current study is limited by its small sample size and qualitative scope, yet it provides an empirical basis for designing multimodal explainable feedback. Future work will implement and validate the proposed architecture through prototype testing with coaches and athletes. Through this approach, the project aims to advance both the scientific and practical frontiers of explainable performance analytics in aquatic sports.

## Figures and Tables

**Table 1:** Summary of Results.

Parameter	Range/Mode	Interpretation
Number of swimmers per session	1–20 (mean ≈10)	System must scale for individual and group use
Importance of explainability	Mean 4.6/5	High priority
Preferred visualisation	75% Video replay with highlights	Visual explanation preferred
Preferred device	87% Tablet/Smartphone	Mobile deployment essential

## Data Availability

The original contributions presented in this study are included in the article. Further inquiries can be directed to the corresponding author(s).

## Abbreviations

The following abbreviations are used in this manuscript:

AI	Artificial Intelligence
XAI	Explainable Artificial Intelligence
IMU	Inertial Measurement Unit
NIRS	Near-Infrared Spectroscopy
SHAP	SHapley Additive Explanations
LIME	Local Interpretable Model-agnostic Explanations
MXAI	Multimodal Explainable AI
DIME	Disentangled Multimodal Explanations
HAR	Human Activity Recognition
CNN	Convolutional Neural Network
GRU	Gated Recurrent Unit
PDP	Partial Dependence Plot
LRP	Layer-wise Relevance Propagation
HIPE	Hierarchical Prototype Explanations
SmO2	Muscle Oxygen Saturation
IBM	International Business Machines (Technology Company)
EO SwimBETTER	Commercial IMU sensor
TrainRed	Commercial NIRS sensor measuring SmO2

## Appendix A. Questionnaire Format

We are developing a coach-facing dashboard for our two projects, DIVE/SWIM-360, a system that integrates wearable sensors, physiology monitoring, and video analysis to provide clear, explainable feedback on swimmer performance. Our goal is to create a tool that supports your coaching by giving you the right information at the right time, in a way that is both practical and easy to use.

The questionnaire consists of some short questions and should take no longer than 8–12 min to complete. All responses will be kept completely private, and we will not ask for any personal information at any stage.

Your input is extremely valuable. By sharing your thoughts and preferences, you will help us design a dashboard that reflects the real needs of coaches and ultimately make DIVE and SWIM-360 as useful and effective as possible.

Before continuing, please confirm that you understand the following: Your responses will be used solely for research and design of the DIVE/SWIM-360 coaching dashboard.

- No personal or identifying information will be collected.
- Participation is voluntary, and you may stop at any time.
- All responses will remain confidential and stored securely.
- By ticking the box below, you give your informed consent to take part in this questionnaire.

Q0. I have read and understood the above information and consent to participate in this questionnaire. (Required to continue)

- Yes (Continue)
- No (Submit Form)

Type: Multiple choice (single answer)

Reason: It is important to obtain consent from a coach and to ensure the coach understands what the form is used for.

### Appendix A.1. General Information

Q1. What level of swimmers do you primarily coach?

- Age-group
- National
- Masters
- Other: ________

Type: Checkboxes (allow multiple selections)

Reason: Helps tailor dashboard insights and feedback complexity to the coaching level, since data needs and feedback styles differ between age groups, national, and masters swimmers.

Q2. Which country do you currently primarily coach in?

Type: Short Answer Text

Reason: Different countries may have different focuses on what to aim for in swimming.

Q3. How many swimmers do you typically monitor per session?

- 1–4
- 5–10
- 11–20
- More than 20

Type: Multiple choice (single answer)

Reason: Determines scalability needs for the dashboard (e.g., number of swimmers tracked simultaneously, level of data aggregation required).

Q4. What type of feedback from a system like our project would be most useful for your coaching? (Tick all that apply)

- Stroke technique (e.g., timing, balance, symmetry, efficiency of movement)
- Physical condition (e.g., fatigue levels, recovery status, breathing/oxygen use)
- Performance metrics (e.g., speed, stroke rate, turns, overall efficiency)
- Injury prevention signs (e.g., early indicators of stress or overuse)
- Other: ________

Type: Checkboxes (allow multiple selections)

Reason: Identifies which performance domains (technique, physiology, performance, injury prevention) should be prioritised in dashboard design and data visualisation.

Q5. Rank the following in order of importance for coaching feedback:

- Stroke efficiency
- Fatigue and recovery
- Injury prevention
- Race performance (speed, times, turns)

Type: Multiple-choice grid with ranking

Reason: Helps prioritise which data the dashboard should highlight most prominently.

Q6. How important is it that the system explains why a certain result or alert was generated?

- 1—Not Important
- 5—Very Important

Type: Linear scale (1–5 for each item in a grid)

Reason: Directly tests the importance of XAI for coaches.

### Appendix A.2. Visualisation Preferences

Example of Video Replay with highlights.

In addition to showing the video, additional information is shown on top, such as the distance to go, etc.

(Example Image Showing Video Replay)

Q7. How important is it that the system explains why a result or alert appears? (1 = Not Important 5 = Very Important)

- 1—Not Important
- 5—Very Important

Type: Linear scale (1–5 for each item in a grid)

Reason: Measures how much value coaches place on interpretability and transparency in visual outputs, guiding how much explanation or justification should accompany system feedback.

Q8. If you could choose only one main type of visualisation to discuss performance, which would help you most?

- Video replay with highlights
- Graphs or trend lines
- Colour-coded visuals (e.g., balance, fatigue)
- Side-by-side comparisons (past vs. elite swimmers)

Type: Multiple choice (single answer)

Reason: Identifies preferred explanation format.

Q9. If you could pick one main screen to use every day, which would you find most useful?

- Summary dashboard (quick overview with key alerts and performance trends)
- Stroke technique view (detailed breakdown of how the stroke is performed)
- Swimmer condition view (fatigue, recovery, and early signs of injury risk)
- Customisable view (you choose what’s shown, depending on the session focus)

Type: Multiple choice (single answer)

Reason: Reveals what coaches want to see first when opening the dashboard.

Q10. Rate how helpful each type of visual would be for your coaching

- Video with highlights (e.g., showing stroke mistakes or movement issues)
- Side-by-side comparisons (e.g., your swimmer vs. an elite swimmer, or vs. their past sessions)
- Graphs and trend lines (e.g., showing changes in speed, stroke rate, or lap times over time)
- Colour-coded visuals (e.g., showing imbalance, fatigue, or weak areas)

Type: Linear scale (1–5 for each item in a grid)

Reason: Gauges the usefulness of visual explanation elements for dashboard design.

### Appendix A.3. Practical Use

Q11. Preferred data format:

- Simple numbers (e.g., stroke rate = 52 spm)
- System interpretation (e.g., “stroke rate below optimal range”)
- Both side by side

Type: Multiple choice

Reason: Determines if coaches want raw data, interpretation, or a mix.

Q12. At first glance, how much information would you like to see?

- Key highlights only (with option for details)
- Mix of highlights + some details
- Full detailed data immediately

Type: Multiple choice

Reason: Determines information density preferences (avoiding overload).

Q13. How important is getting feedback during a training session compared to reviewing it afterwards?

- Essential (I want feedback immediately while the swimmer is training)
- Important, but post-session review is enough for me.
- Not necessary

Type: Multiple choice

Reason: Helps set system requirements for real-time vs offline dashboards.

Q14. Which devices would you most likely use the dashboard on?

- Laptop/PC
- Tablet
- Smartphone
- Poolside large screen (visible to swimmers and coaches)

Type: Checkboxes

Reason: Informs responsive design and context-of-use requirements.

Q15. How often would you realistically use such a dashboard during training or competition preparation?

- Every training session
- A few times a week during training
- Once a week during training
- Less than once a week during training
- Primarily before competitions (for race preparation and analysis)

Type: Multiple choice

Reason: Helps scope usage frequency and design priorities.

### Appendix A.4. Technique and Biomechanical Feedback

In this section, we are looking to identify the key features which should be identified for swimmers when providing feedback on their technique across all strokes, turns, underwater streamline and dives.

Q16. For each of the following, how important would this type of feedback be across any stroke? (1 = Not Important 5 = Very Important)

- Body alignment and rotation
- Kick timing and symmetry
- Breathing timing/head lift
- Arm pull depth/efficiency
- Glide or underwater phase duration
- Start, turn, and push-off efficiency

Type: Linear scale (1–5 for each item in a grid)

Reason: Determines which biomechanical metrics are most relevant across strokes, ensuring the system focuses on the feedback elements that have the highest practical value for coaches.

Q17. If you could identify only three key elements to monitor in the dashboard, what would they be?

Type: Paragraph (open text)

Reason: Helps identify the highest-priority data points for inclusion, revealing which metrics coaches value most for everyday decision-making and training impact.

Q18. What is the biggest challenge you face when analysing swimmer performance without technology (e.g., video, sensors, data tools)?

Type: Paragraph (open text)

Reason: Gathers qualitative feature requests directly from coaches.

Q19. What would make DIVE/SWIM-360 most useful in your day-to-day coaching?

Type: Paragraph (open text)

Reason: Identifies the main gaps that SWIM-360 could fill.

## References

[1] Gulshad S., Long T., van Noord N. Hierarchical Explanations for Video Action Recognition. Proceedings of the 2023 IEEE/CVF Conference on Computer Vision and Pattern Recognition Workshops (CVPRW).

[2] Tjoa E., Guan C. (2021). A Survey on Explainable Artificial Intelligence (XAI): Toward Medical XAI. IEEE Trans. Neural Netw. Learn Syst..

[3] Rodis N., Sardianos C., Radoglou-Grammatikis P., Sarigiannidis P., Varlamis I., Papadopoulos G.T. (2024). Multimodal Explainable Artificial Intelligence: A Comprehensive Review of Methodological Advances and Future Research Directions. IEEE Access.

[4] Arya V., Bellamy R.K.E., Chen P.-Y., Dhurandhar A., Hind M., Hoffman S.C., Houde S., Liao Q.V., Luss R., Mojsilović A. (2020). AI Explainability 360: An Extensible Toolkit for Understanding Data and Machine Learning Models. J. Mach. Learn. Res..

[5] Saha A., Gupta S., Ankireddy S.K., Chahine K., Ghosh J. Exploring Explainability in Video Action Recognition. Proceedings of the IEEE/CVF Conference on Computer Vision and Pattern Recognition.

[6] Zhi Y., Tong Z., Wang L., Wu G. MGSampler: An Explainable Sampling Strategy for Video Action Recognition. Proceedings of the 2021 IEEE/CVF International Conference on Computer Vision (ICCV).

[7] Mahmud S., Tanjid Hasan Tonmoy M., Kumar Bhaumik K., Mahbubur Rahman A.K.M., Ashraful Amin M., Shoyaib M., Asif Hossain Khan M., Ahsan Ali A. (2020). Human Activity Recognition from Wearable Sensor Data Using Self-Attention. Frontiers in Artificial Intelligence and Applications.

[8] Ma H., Li W., Zhang X., Gao S., Lu S. (2019). AttnSense: Multi-Level Attention Mechanism For Multimodal Human Activity Recognition. Proceedings of the Twenty-Eighth International Joint Conference on Artificial Intelligence.

[9] Lattanzi E., Calisti L., Capellacci P. (2023). Lightweight Accurate Trigger to Reduce Power Consumption in Sensor-Based Continuous Human Activity Recognition. Pervasive Mob. Comput..

[10] Vijay Arya (2025). Trusted-AI/AIX360. https://github.com/Trusted-AI/AIX360.

[11] Sandamal K., Arachchi S., Erkudov V.O., Rozumbetov K.U., Rathnayake U. (2024). Explainable Artificial Intelligence for Fitness Prediction of Young Athletes Living in Unfavorable Environmental Conditions. Results Eng..

[12] Navakauskas D., Dumpis M. (2025). Wearable Sensor-Based Human Activity Recognition: Performance and Interpretability of Dynamic Neural Networks. Sensors.

[13] LinLin H., Sangheang L., GuanTing S. (2024). CAM-Vtrans: Real-Time Sports Training Utilizing Multi-Modal Robot Data. Front. Neurorobot..

[14] Almasi S., Bahaadinbeigy K., Ahmadi H., Sohrabei S., Rabiei R. (2023). Usability Evaluation of Dashboards: A Systematic Literature Review of Tools. BioMed Res. Int..

